# Academic Achievement in Spanish Secondary School Students: The Inter-Related Role of Executive Functions, Physical Activity and Gender

**DOI:** 10.3390/ijerph18041816

**Published:** 2021-02-13

**Authors:** Elena Escolano-Pérez, Marta Bestué

**Affiliations:** 1 Faculty of Education, University of Zaragoza, 50009 Zaragoza, Spain; 2 Faculty of Human Sciences and Education, University of Zaragoza, 22003 Huesca, Spain; mbestue@unizar.es

**Keywords:** academic achievement, executive functions, physical activity, gender, academic year, adolescent

## Abstract

There is a growing interest in determining which variables contribute to students’ academic performance, since this performance is associated with their wellbeing and with the progress of the nation. This study analyzed whether different variables (executive functions and physical activity levels, gender and academic year) of 177 Spanish Compulsory Secondary School students contributed to their academic performance. The Behavior Rating Inventory of Executive Function 2 (BRIEF-2), Physical Activity Questionnaire for Adolescents (PAQ-A) and an ad hoc questionnaire were used to determine the students’ executive functioning, physical activity level, gender and academic year, respectively. Students’ grades were considered to be indicators of their academic achievement. Seven multiple linear regression models were constructed using the R computing language to examine the association between academic achievement (considered in each of the 5 subjects: Language, Mathematics, Geography and History, English and Physical Education; the mean of the instrumental subjects—Language and Mathematics—and the mean of all the subjects) and the independent variables. The results indicated that executive functions, physical activity and gender contributed to academic performance, but academic year did not. This suggests that students with good executive functions, who perform physical activity and are female, would have better academic achievement. This information should be considered when designing interventions to improve student academic achievement.

## 1. Introduction

Increasing student academic achievement (AA) is a major goal for all governments since level of education has been closely related to personal wellbeing and the nation’s social and economic progress [1]. However, this is a complex issue, beginning with the definition and operationalization of the construct.

Although numerous definitions of AA have been created, it is generally considered to be the level of knowledge, skill and attitudes demonstrated by a student in a certain area, subject or course, as compared to the norm. Typically, AA is expressed through the qualifications provided by teachers. This is based on an evaluation that reflects the level of student compliance with the goals, achievements and objectives of the class or course during the education process [2]. Although the assessment of AA through teacher qualifications has been questioned by some authors due to the possible influence of teacher subjectivity, it continues to be the most commonly used indicator of AA [3]. In fact, in many countries (including Spain, where this study was conducted), it continues to be the legally established assessment mode [4].

Increasing student AA has become a major goal in certain countries, given the low levels of student performance, especially for certain education levels. This is the case for Spain. When compared to other countries of the Organization for Economic Cooperation and Development (OECD) and the European Union (EU), Spain is lower in secondary education level (corresponding to level 2 of the International Standard Classification of Education—ISCED—approved by the United Nations Educational, Scientific and Cultural Organization—UNESCO—in 2011) and has been found to have a high rate of students who repeat a year and a high number of students who fail to complete this education level. Furthermore, it has been suggested that those Spanish students who do manage to complete this level of studies tend to have a lower mean AA when compared to students from other countries of the OECD and EU [5].

In Spain, lower secondary education (ISCED 2) is referred to as Compulsory Secondary Education. It is the last mandatory education phase and consists of 4 academic years, corresponding to the ages of 12 to 16. It is a free and fundamental education period that allows students to obtain a basic level of knowledge and skills that are considered necessary for adult life. After completing it, students obtain a Lower Compulsory Secondary Education Certificate. This is the first official certificate that they receive, and it permits them to continue their studies down either an academic or vocational route or allows entry to the labor market. This is the education level that has the highest academic failure rates (an aspect also occurring in other European countries). Students do not acquire the knowledge and skills that are necessary to advance to the next level and, eventually, they drop out [6]. In 2018, for example, the rate of students who repeat a year at the end of the mandatory education phase in Spain was 28.65%—considered by the OECD to be an alarming rate [7]—while in the EU, it was 13% and, in the OECD, 11.45%. As for early school dropout (understood as the percentage of individuals aged 18 to 24 that failed to achieve this ISCED 2 education level, or, upon achieving it, did not continue with their studies), in Spain in 2019, it was 17.3%. The mean for EU countries was 13% [5]. This dropout is considered “early” since, for the purposes of personal development, understanding modern society or entering the labor market, and all other practical purposes, it is necessary to achieve a higher degree of education than this.

Reducing rates of early school dropout was one of the five main objectives of the Europe 2020 Strategy created in 2009 to promote EU economic recovery. Specifically, the goal was to reduce the early school dropout percentage to under 10%. In Spain, given the high dropout rate (30.9% in 2009), the proposal was to reduce the percentage to under 15%. Despite having been dramatically reduced over the past 10 years (going from 30.9% in 2009 to 17.3% in 2019), it is still yet to reach this desired 15% [5]. This situation is also being aggravated nowadays by the COVID-19 pandemic. School closures due to COVID-19 point to a reduction in average learning levels for all students. The share of children in lower secondary education below the minimum proficiency level will rise. It could provoke massive dropouts. More exactly, school closures could imply that more than 25% of students do not reach the necessary skills to participate effectively and productively in society and in future learning. Statistical simulations on developing countries participating in the OECD’s Program for International Student Assessment (PISA) suggest that, without compensatory or remediation actions, a 3-month school closure might result in a one-third loss of learning. In terms of PISA test score, this reduction means a decrease of 8 points, which is the current difference between Spain and the OECD average [8,9].

These data justify the fact that currently, in the 2030 Agenda for Sustainable Development adopted by the United Nations [10], and in the OECD Future of Education and Skills 2030 project [11], early school dropout and learning outcomes continue to be a major cause of concern. Therefore, the goal proposed for 2030 is that all students complete quality primary and secondary education, which results in the establishment of relevant and effective learning outcomes [10]. A solid educational foundation established in primary and secondary education levels may have a great influence on the individual’s ability to advance in life. This suggests that failing to complete secondary education, or having inadequate academic results at this level, may have negative consequences for individuals and society, from both a general wellbeing and labor market perspective.

In light of the above, this work examines AA in a sample of Spanish students in Compulsory Secondary School Education. More specifically, it attempts to determine whether certain student variables (to be explained in greater detail below: executive functions, physical activity level, gender and academic year) have influence. Determining which variables contribute to the AA of students will help improve the design of intervention strategies. The resulting benefits on health and personal wellbeing are abundant [12,13], and therefore, have an impact on the nation’s economic and social progress [1,14]. This is due to the clear fact that today’s students will be tomorrow’s leaders [15,16].

### 1.1. Variables Influencing the AA of Students

The variables associated with student scholastic success or failure are extensive and there are many distinct types: student (for example, the level of cognitive, psycho-motor or emotional development), family-based (the education level of parents, family income level, shared activities between parents and children and frequency of these activities, etc.) and educational (the teacher’s educational methodology, classroom environment, etc.) [17].

These numerous variables are inter-related and are complex in their detection and identification. Therefore, studies are necessary to simplify their investigation, focusing on the study of just some of these variables and not all at once [17]. This work examines four variables: the student level of executive functions, student level of physical activity, gender and academic year. These variables were selected based on their relevance to the topic at hand [18,19].

Studies have usually examined AA in general terms, without considering the distinct academic areas forming the school curriculum. The few studies that have looked at classes separately have typically considered Language and Mathematics (instrumental subjects), omitting the other classes. To overcome this gap, the AA of several subjects was taken into account in this study. Specifically, the AA obtained for each of the five subjects studied in all of the Compulsory Secondary Education courses (Language, Mathematics, Geography and History, English and Physical Education) was considered. Furthermore, Instrumental AA (arithmetic mean AA obtained in Language and Mathematics) and Overall AA (arithmetic mean AA obtained in the five subjects mentioned) were also considered.

Below, we consider each of the conditioning variables of AA that were analyzed in this study: executive functions, physical activity, gender and academic year. As previously mentioned, we were interested in determining their association with AA in a sample of Spanish Compulsory Secondary Education students.

#### 1.1.1. Executive Functions (EF)

Executive functions (EF) are a broad set of higher order cognitive and affective processes that allow us to direct, coordinate and regulate our thoughts, emotions and behaviors in order to achieve our objectives. Thus, they are crucial for daily functioning. They are especially necessary in novel or demanding situations that require a rapid and flexible adjustment of behavior in response to the changing environmental demands. They allow us to resolve problems that we have never been faced in the past, potentially anticipating solutions and their consequences [20,21].

The EF processes supporting goal-directed behavior are different across the distinct contexts [21]. Differentiation is made between the cool and hot EF (or the cognitive and socioemotional EF). Cool EF refers to those processes required in motivationally or affectively neutral contexts, i.e., abstract, decontextualized contexts. The cool EF components include working memory (the ability to temporarily store and manipulate information), inhibition (suppressing prepotent responses, halting ongoing process and avoiding distractor stimuli to select the relevant one) and cognitive flexibility (mentally changing between different tasks or sets), in addition to other more complex ones such as planning (choosing and implementing a sequence of actions to achieve a pre-specified goal) [20,21]. Hot EF refers to those processes that are required to perform goal-directed tasks in motivational or affectively salient contexts, e.g., meaningful, self-relevant reward or punisher contexts. Some of the hot EF components are, for example, emotional regulation (the capacity to manage one’s own emotional responses) or the ability to delay gratification (resisting the temptation of immediate compensation and waiting for a more valued, subsequent compensation) [21,22].

The development of EF skills is a complex and protracted process. Although the different EF components display distinct developmental trajectories, generally speaking, the EF begin to develop at the end of the first year of life. During childhood and adolescence, they undergo major development, reaching levels of maturity in early adulthood (approximately 20 years of age). From this moment until approximately 50 years of age, they remain relatively stable, showing a decline from this latter age. Therefore, it may be said that the development of the EF during the lifecycle follows an inverted U-shaped curve [20]. All of the changes taking place in the EF are associated with major brain changes. Of these brain changes, those which occur in the prefrontal cortex during adolescence are especially significant, such as synaptic pruning or the cutting of weak connections, a decline in grey matter, and especially, the increase in white matter. All of these mean that adolescence is a developmentally sensitive period for EF [22,23].

Today, the study of EF has become an area of special interest for researchers, given its relevance and implications in many areas of life. EF are crucial for daily functioning. They are not only important for cognitive development and change, but also for personal and social development. Therefore, they are related to academic, labor, social, and personal adjustment and success, as well as health and quality of life [20,23].

In the academic sphere, EF serve as predictors of AA, possibly even better than IQ [24]. In the classroom and learning context, students are required to use their EF. Student achievement requires the ability to recall instructions and represent the goal of the lesson (working memory), attend to the important features of the lesson and stay on task, ignoring other stimuli that may be more appealing (inhibition), and switching their attention between tasks or rules (cognitive flexibility), among other aspects. This suggests that cool EF may play a significant role in AA. Furthermore, the extent to which students can regulate their emotions (for example, their test anxiety) and attend to academic content may depend, in large part, upon hot EF abilities [25]. Therefore, the classroom and learning environment demand the use of both hot and cool EF, and this means that hot and cool EF may be critical skills for children’s learning and AA.

The majority of works analyzing the relationship between EF and AA focus on preschoolers and school-aged children, with very few works considering adolescents [26]. Therefore, the association between these two variables during this period remains poorly understood [22,27]. This study attempts to eliminate this information gap, containing an integral view of EF which includes hot and cool components, since most studies investigating EF only measure cool components [22]. In addition, in this study, as previously mentioned, we consider both AA in general as well as in the different individual classes and in the instrumental ones. We are unaware of other studies that consider the relationship between EF and AA in distinct classes, with the exception of Language and Mathematics. We hope that this work will contribute to an increased knowledge of this topic.

#### 1.1.2. Physical Activity (PA)

Another variable that was considered in this study was physical activity (PA). This refers to any body movement produced by the skeletal muscles that requires energy consumption [28].

There is much evidence regarding the physical, cognitive and psychosocial benefits of carrying out regular PA. These benefits also affect AA. Many studies have shown that engaging in regular PA is closely related to good AA [29,30]. However, some studies have failed to find any type of relationship between these variables [31], and other works have even found an inverse relationship [32]. The distinct methodologies used to quantify both PA (considering intensity, duration, type, etc.) as well as AA (using qualifications, standardized tests, observation, etc.), in addition to differential characteristics of the studied samples, may help explain discrepancies between results of the studies [33].

The mechanisms by which PA may offer benefits to AA are not well-known [33,34,35]. There are two main theories or perspectives that explain how PA can be beneficial to cognitive processes and, therefore, how it may be associated with AA: the neurophysiological perspective and the psychosocial perspective [30,36]. The main argument of the neurophysiological perspective to explain the relationship between PA and AA is that PA offers direct, positive effects on the brain and nervous system. PA produces increased brain functioning by increasing blood flow, boosting glucose and lipid metabolism, resulting in a higher capacity for concentration and improved cognitive capacities. PA also increases the level of several growth factors such as brain-derived neurotrophic factor (BDNF) [30]. BDNF has been found to improve neural survival, neural maintenance, neurogenesis, synaptic plasticity, long-term potentiation and neurotransmitter release. All of these factors are involved in the maintenance and plasticity of the brain’s structure and function, making it more efficient and adaptive, leading to improved memory, attention and learning. Increasing BDNF is also associated with an increase in the volume of the hippocampus, a brain area that is highly related to memory [35]. The psychosocial perspective indicates that participation in PA with peers improves social skills such as cooperation and respect for the rules and may have a positive influence on AA [33,36]. PA is also positively associated with mental health components, such as self-esteem, resilience capacity, tolerance to adversity, positive acceptance of changes and emotional wellbeing. This may also contribute to an increased motivation and may have implications on studies, as well as their better management and on the feelings associated with these studies. All of this may result in an improved AA. Therefore, PA may also indirectly improve cognition and AA [33,34,35,36,37,38]. The explanations offered by both perspectives are not exclusionary and although they all contribute to an improved knowledge of the relationship between PA and AA, the understanding of these relationships is still in an initial phase, with many issues remaining to be clarified and known [34].

For example, there are works which, based on past results, reveal associations between PA and AA, and have gone further, seeking to determine if the benefits of PA occur in only one academic area, in various areas, or in all areas. However, the results between the studies are quite disparate. Some authors, such as Singh et al. [35], have concluded that there is strong evidence for the beneficial effects of PA on Mathematics, but find inconclusive evidence for Language performance. On the other hand, Hillman et al. [39] found associations between PA and reading comprehension but not with spelling or arithmetic. Other results are distinct, failing to find relationships between PA and Mathematics or with Language, although finding a connection with Physical Education [40]. Other authors, however, have found that the relationships between PA and AA take place in multiple academic areas. Along this line, many works have found relationships between PA and mathematics and reading, in addition to relationships with composite scores for AA [41,42].

Given this variety of results, our study attempts to offer additional information. It aims to determine the extent to which PA, in addition to other variables of interest, contributes to the student’s AA, including AA in classes that have yet to be examined by researchers. It also considers AA in the instrumental subjects and overall AA.

#### 1.1.3. Gender

Gender is another variable of interest in our study. Generally speaking, past studies have affirmed that there is a trend by female students to obtain better academic scores than their male peers [22,43]. Gender has even been considered to be a predictor of performance [44]. The data and statistics provided by distinct organisms and national and international entities have also revealed these gender-based differences. In addition, these differences may already be detected during childhood, increasing during the period of adolescence [11,45].

In Spain, many indicators suggest differences in the AA of Compulsory Secondary Education students based on gender [45]. For example: (1) In 2019, the percentage of dropout was clearly lower for females (13.0%) as compared to males (21.4%). (2) The percentage of students that were promoted to the following academic year was higher in girls as compared to boys: on average, 89.93% of the females were promoted to the next academic year, compared to only 85.15% of the males.

This series of figures and indicators, representing the gender differences existing in Spain in the AA of adolescent students, is representative of what takes place in countries forming part of the OECD and the EU [11]. Although differences between countries exist in the size of the gender gap, this gap continues to favor females.

If considering academic performance, not general performance but rather that of distinct academic areas or classes, the difference between genders has certain nuances, given that boys have better performance in certain areas than girls. Girls tend to show better academic achievement in classes related to language and social areas, while boys do better in mathematics and science. These results are found in studies [46] as well as in international assessments carried out by organizations such as the OECD’s PISA [7]. PISA has consistently found that girls outperform boys in reading while, to a lesser extent, boys outperform girls in mathematics, on average across all participating countries and economies. In science, the differences found in favor of boys are inexistent or very small. Gender disparities in achievement are a matter of considerable concern, since they may have long-term consequences for the children’s personal and professional future [7].

Scientific debates regarding gender differences in AA have highlighted several explanations, which are now considered to be not excluding. Early research on gender differences in cognitive abilities and AA focused primarily on biologically based explanations, including the contribution of hormones. Although both the structure and the function of the brain is remarkably similar in females and males, region-specific structural and functional sex differences exist. These differences are associated with changes in sex hormone (mainly testosterone and estrogen) exposure during puberty. The action of sex hormones creates different neural networks and biochemical processes in the brains of men and women, affecting certain cognitive capacities and possibly having effects on AA. In terms of cognition, testosterone facilitates the processing of certain types of spatial information. This may explain the fact that men (having higher levels of testosterone than women), on average, have an advantage in visual memory, visuospatial ability and math. Women are found to excel in language tasks such as fluency, vocabulary, verbal memory, grammar and verbal learning, which are associated with higher levels of estrogen [46,47]. Over recent decades, the explanations have extended to include sociocultural factors, such as differences between boys’ and girls’ socialization experiences. Boys and girls may be exposed to distinct experiences (both in the family and school and social contexts) that are rooted in gender stereotypes. That is, based on the cultural norms for masculinity and femininity. Gender stereotypes include expectations for a particular gender, as well as the attribution of abilities in specific domains, leading to teachers having distinct patterns of dynamics and interactions for boys and girls, thereby affecting their AA [48]. Some stereotypes, such as the one suggesting that men are better than women in spatial and mathematical abilities and that professions and subjects in Science, Technology, Engineering and Mathematics (STEM) are “male” domains, continue to be believed in many contexts. These stereotypes can reduce female’s motivation, interest, learning behaviors, skills and consequently, their AA, in domains that are culturally viewed as “masculine”. However, boys’ performance can benefit from such stereotypes [49].

Regarding the potential gender differences in academic performance in areas other than Language, Mathematics and Science, existing data are scarce; generally speaking, however, they tend to support these differences. González Hernández and Portolés Ariño [50] analyzed gender differences in Spanish Compulsory Secondary Education students in the following classes: English, Social Studies and Physical Education, in addition to Language, Mathematics and Sciences. The authors found that girls obtained higher mean grades in all areas except for Physical Education, in which boys obtained higher grades. However, these differences are only statistically significant in Language, English and Physical Education (not in Science, Social Studies and Mathematics).

More research is necessary to contrast these data and learn more about the influence of gender on AA in areas other than Language and Math. This work attempts to serve as a contribution in this sense.

#### 1.1.4. Academic Year

Both past research as well as the data from distinct national and international studies indicate that the student’s academic year affects their AA. When focusing on the four school years of Compulsory Secondary Education (the educational phase of interest in this study), Hernando et al. [51] found a statistically significant and negative relationship between the Spanish student’s AA and their year of study at that time. It was found that the student’s AA decreased as the student’s Compulsory Secondary Education year advanced. Studies conducted in other countries also revealed this descending trend in AA as the academic year of the student advanced [52].

Data provided by the Spanish Ministry of Education and Vocational Training (SMEVT) [45] followed in this same direction. For example, the percentage of students that passed their year of Compulsory Secondary Education decreased as the Compulsory Secondary Education year advanced, finding the following rates for each year of study (from 1st to 4th): 89.7%, 87.5%, 87.0% and 85.8%.

Different results were provided by González Hernández and Portolés Ariño [50]. Although these authors also found some significant differences in AA for students in the four years of Compulsory Secondary Education, they did not find a stable pattern for such differences. That is, their results, unlike those of Hernando et al. [51] and those from the SMEVT data [45], did not show that AA decreased as the course year increased. The difference between these results may be due, among other things, to the means by which AA was measured in each study. González Hernández and Portolés Ariño [50] used academic grades from 6 classes (Math, Spanish language, English, Physical Education, Social Studies and Science) in four consecutive quarters, expressing the AA in each class through the mean grade of the same. Hernando et al. [51], however, assessed AA through an item in which the student was asked for the grades that they obtained at the end of the previous year, offering them six response options (1 = several fails: 3 or more; 2 = a few fails: 1 or 2; 3 = barely passed or just barely; 4 = passed and some pass with distinction; 5 = distinction and some outstanding grades and 6 = outstanding in almost every class). The figures provided by the SMEVT [45] do not consider the specific grades obtained for the classes, but rather, are based on other parameters such as passing the academic year (regardless of doing so while passing all classes or not, and not considering the specific grades received in each class).

Even though, as previously mentioned, these methodological differences may help explain the discrepancy between the results obtained, more research is necessary to offer additional knowledge on this area. This study aims to contribute in this way.

### 1.2. Aim

In light of the above, and in an attempt to eliminate the previously mentioned gaps, this study sought to answer the following research questions: Do EF, PA, gender and academic year contribute to Spanish students’ AA in Compulsory Secondary School Education? Do these variables contribute in the same way when AA is assessed in each of the subjects as when instrumental AA or overall AA is assessed?

The objective was to analyze whether the four previously cited variables (EF, PA, gender and academic year) contribute to AA in a sample of Spanish Compulsory Secondary Education students. This was to be done through considering different AA measures: AA in each of the five common mandatory classes that are studied throughout this educational phase (Language, Mathematics, Geography and History, English and Physical Education), the AA based on the mean of the two instrumental subjects (Language and Mathematics) and overall AA (mean of the 5 classes, collectively).

It was hypothesized that EF, PA, gender and academic year would be associated with: (1) Language AA, (2) Mathematics AA, (3) Geography and History AA, (4) English AA, (5) Physical Education AA, (6) Instrumental AA and (7) Overall AA. Specifically, it was hypothesized that not presenting EF problems, showing a higher level of PA, being male and studying less advanced academic years would contribute to a higher AA in Mathematics and Physical Education. Further, it was hypothesized that not having EF problems, showing a higher level of PA, being female and studying less advanced academic years would contribute to a higher Language, Geography and History, English, Instrumental and Overall AA.

To determine these associations, relevant information is provided so as to design and implement interventions and educational practices that focus on the most relevant variables for AA. At the same time, this would support the student’s academic success, promoting their wellbeing and contributing to advance the country [1,14]. Assuring the educational success of its citizens helps ensure the progress of the country [1,14,15,16].

## 2. Materials and Methods

### 2.1. Participants

This cross-sectional study involved 177 Spanish students in the Compulsory Secondary Education phase (55.37% male, 46.63% female: mean age = 13.7, standard deviation (SD) = 1.35). They were all recruited from the same charter school. The students were in the 4 years making up Spanish Compulsory Secondary Education: 51 (28.81%) were in their 1st year, 44 (24.86%) were in their 2nd year, 36 (20.34%) were in their 3rd year and 46 (25.99%) were in their 4th year. Their socio-economic level was average–high (according to information provided by the school’s management team).

The inclusion criteria of the sample were: (a) having Spanish as maternal language, (b) receiving the informed consent of the parents/guardians who authorized participation of their child in the study and (c) having the voluntary declared participation of the participant. The exclusion criteria were: (a) having intellectual disability, (b) having learning difficulties and (c) having sensory, psychiatric, or neurological problems. Information referring to the inclusion criterion (b) and information referring to the three exclusion criteria were provided by the school’s management team.

Participants were treated according to the rules of the Declaration of Helsinki of 1975—revised in 2013—and Spanish Organic Law 15/1999 of December on Protection of Personal Data (Spanish Government, 1999), published in the Official State Gazette n_ 298, 14 December. Research was endorsed by the Education Doctoral Program Academic Commission of Zaragoza University and by the management team of the school attended by the participants. In addition, as previously indicated, informed consent was received from all parents of the participants as well as the voluntary and manifest consent from each of the participants.

### 2.2. Instruments

#### 2.2.1. Instruments for Data Collection

To assess the EF of the students, the Behavior Rating Inventory of Executive Function 2 (BRIEF-2) was used in its school version, adapted for the Spanish population [53]. It consists of a battery of 63 items to be responded to on a Likert-like scale of frequency (never, sometimes, frequently) by the student’s teacher or any other adult having prolonged or frequent contact with them in the school setting. BRIEF-2 is made up of nine scales grouped in three major indices: (1) Behavioral Regulation Index: indicates the difficulty a student has to regulate and monitor their behaviors effectively. It is composed by the two following scales: Inhibition: informs about the students’ problems to control their impulses (i.e., “Is fidgety”), and Self-Monitor: problems self-monitoring their own behavior (i.e., “Is unaware of how his/her behavior affects or bothers others”). (2) Emotional Regulation Index: indicates the difficulty degree to regulate the emotional responses, specifically to changing situations. It is composed by the following scales: Shift: presence of problems to make transitions/change the attentional focus if the activity requires to solve issues in a flexible way (i.e., “Has trouble getting used to new situations (classes, groups, friends, etc.)”), and Emotional Control: issues to control their emotional responses (i.e., “Has explosive, angry outbursts”). (3) Cognitive Regulation Index: refers to the difficulty degree to control and manage their own cognitive processes and solve problems effectively. It is composed by five scales: Initiate: problems to start tasks autonomously or to generate new ideas, responses or strategies in solving problems (i.e., “Has trouble getting started on homework or tasks”), Working Memory: indicates difficulties in keeping information in mind during enough time to execute an activity (i.e., “Forgets what he/she was doing”), Plan/Organize: evaluate the appearance of problems to anticipate future situations, order and prioritize information, set objectives and sequence the necessary steps to achieve them (i.e., “Underestimates time needed to finish tasks”), Task-Monitor: grade of difficulty to check their own work, assess the execution and ensure the achievement of the objective (i.e., “Makes careless errors”) and Organization of Materials: evaluates the presences of problems to keep the study, work, playing and their own things areas organized (e.g., desk, wardrobe, backpack, bedroom) (i.e., “Cannot find things in room or school desk”). These three indices, in turn, are summarized in the Global Executive Function Index, the index used in this study. A high score indicates the presence of cool and hot executive function problems, that is, difficulty regulating one’s behavior, emotions and cognitive processes and difficulties in effectively solving problems. The instrument has good psychometric properties in Spanish samples [53]. The internal consistency value (Cronbach’s Alpha) in this research was adequate (α = 0.834).

To measure the PA level, the Physical Activity Questionnaire for Adolescents (PAQ-A) was used in the version validated for Spanish adolescents [54]. It consists of 9 questions (to be responded to on a 5-point Likert-like scale) that assess distinct aspects of the PA carried out over the past 7 days. Specifically, it assesses the physical activity performed by the adolescent over the last 7 days, during their free time, in their Physical Education classes and at distinct times (lunch, afternoons and evenings) of the class day, as well as over the weekend. The questionnaire also assesses the proposed level of PA, to better describe the physical activity carried out during the week and the frequency with which the adolescent carries out PA every day of the week. The final score was obtained by calculating the arithmetic mean of the scores obtained on these 8 questions. Question 9 offered information as to whether the adolescent was sick or if there was any circumstance preventing them from carrying out PA that week. The final result has a value from 1 to 5 which permits the establishment of a PA scale. The PAQ-A is found to have adequate reliability and reasonable validity to assess PA in Spanish adolescents [54]. Past studies have indicated that the result provided by this questionnaire is similar to that obtained via a more complete study of PA using techniques such as accelerometry [55]. As such, this makes it one of the most widely used questionnaires to assess PA in adolescents and one that has been validated in multiple languages. In our study, the internal consistency value (Cronbach’s Alpha) was 0.775 and was considered to be an adequate value. This value is similar to the one obtained by Martinez-Gómez et al. [54] in the instrument validation for Spanish adolescents.

To determine the student’s gender and academic year, an ad hoc questionnaire was created, made up of only 2 questions: one referring to gender and the other to year. The response should be indicated, selecting one of the possible alternatives offered: male or female for the gender question, and 1st, 2nd, 3rd or 4th for the academic year question.

To determine the AA in each class of interest (Language, Mathematics, Geography and History, English and Physical Education), the arithmetic mean was calculated for the qualifications obtained by each student in each class throughout the school year. To do so, the management team provided the quarterly grade of each participant in each class for each of the three quarters of the Spanish school year. With these grades, the research team calculated the final mean of each class. The grades, based on the Spanish system, were always given on a system of 0 to 10, with 0 being the lowest grade and 10 being the highest (with ≥5.0 being a passing grade). To determine the mean AA in the instrumental classes (Instrumental AA), the arithmetic mean was calculated for the AA obtained in Language and Mathematics. To determine the Overall AA, the arithmetic mean was calculated for the AA obtained in the five classes of interest. The arithmetic mean of the grades given by the student’s teacher was considered to be the indicator of the AA of the same, a common practice used in many studies [3,43].

#### 2.2.2. Data Analysis Software

To process the data (more specifically, to check our dataset for missing data), the ‘VIM’ package [56] of R computing language version 3.6.1 (Vienna, Austria) [57] was used.

To perform an analysis that would respond to our study objective (determining whether EF, PA, gender and grade were associated with AA), the R computing language version 3.6.1 [57] was used. Specifically, the following packages were used: ‘Stats’ [57], ‘QuantPsyc’ [58], ‘GGally’ [59], ‘ggplot2’ [60], ‘gridExtra’ [61], ‘lmtest’ [62], ‘car’ [63], ‘corrplot’ [64] and ‘base’ [57].

### 2.3. Procedure

The research team held an informative meeting with the school’s management team. Subsequently, the team sent an informative letter to the parents and legal guardians of all potential participants, offering them the possibility of holding an informative personal meeting if they so desired. Together with the letter, the informed consent form was sent, requesting its return to the management team through the children.

To determine the level of difficulties in EF, the school guidance counselor (who knows the participants well, given the frequent seminars held and the individual and group activities carried out with them), during March 2019, completed the BRIEF-2 School version for each of the participants. The mean time spent on this was approximately 10 min/assessed student. The computerized version was used as well as its online automated correction.

Data collection on the students’ PA level as well as information on gender and academic year was carried out in the classroom of each group-class and during the regular period that was used for coaching during the month of March 2019. The counselor was the individual entrusted with carrying out this collection. After the students agreed to their voluntary participation in the study, each student was given a PAQ-A, as well as the referred gender/year questionnaire. They were informed of how to respond to the questionnaires and any doubts were clarified. Subsequently, each student completed the questionnaires. The mean time spent by the students was approximately 15 min.

At the end of the school year (June 2019), the school provided the mean grades of each participant in each of the three quarters for each of the 5 classes of interest: Language, Mathematics, Geography and History, English and Physical Education. Using this information, the corresponding mean grades were calculated as previously described (Section 2.2.1). In this way, the AA was obtained in each class: Instrumental AA (mean performance obtained in the instrumental classes of Language and Mathematics) and the Overall AA (mean performance on the 5 classes).

### 2.4. Data Analysis

Before performing the analysis, and in order to successfully manage the data, the existence of missing data was examined. To do so, the aggr() function from the ‘VIM’ R environment package was applied. This allowed us to determine that there was no missing data.

Then, in accordance with our objective, a Multiple Linear Regression (MLR) analysis was performed to examine the effects of EF, PA, gender and academic year on AA. The R version 3.6.1 was used. Below, the different steps carried out are detailed.

(1) Based on the study objective, seven MLR models were created to examine how distinct variables influence AA in different areas. Each of these 7 models were created, based on one of the seven measures of interest referring to AA, as the dependent variable. That is, AA in the following classes: (1) Language, (2) Mathematics, (3) English, (4) Geography and History, (5) Physical Education, (6) as well as Instrumental AA (i.e., the average AA of Literature and Mathematics) and (7) Overall AA (i.e., the mean of the AA obtained in the 5 classes). As independent variables, the same variables from the 7 models were considered. In line with our study objective, these variables were: EF, PA, gender and academic year. From R ‘Stats’ package, lm() function was applied to build the MLR models.
(2) A mixed stepwise regression [65] was performed to select the most useful explanatory variables. The Akaike Information Criterion (AIC) [66] was used as a statistical criterion to compare the different possible models and to decide which was the best fit for the data. The preferred model has the lowest value in the AIC. The step() function, included in the R ‘Stats’ package, was applied.
(3) Standardized regression coefficients (β) were calculated. They allow for the contrasting of the independent variables included in the model in order to determine which are more closely related to the dependent variable. For this, the lm.beta() function was applied, available in the ‘QuantPsyc’ package.
(4) Different strategies were applied to verify that the assumptions of the MLR analyses were met [67,68].
  - The linearity assumption (i.e., a linear relationship exists between the dependent and the independent variables) was graphically tested examining: (1) The dispersion diagrams between the dependent and the independent variables. The points should tend to group together around a diagonal line. The ggpairs() function in ‘GGally’ package was used. (2) The dispersion diagrams of the residuals of the model against each of the independent variables. If the residuals are randomly dispersed around the 0 line, the linearity assumption is met. We used the ggplot() function—available in the ‘ggplot2’ package—and the grid.arrange() function—in the ‘gridExtra’ package.
  - Normality of error term distribution, also called normally distributed errors or normality of residuals [68], implies that the residuals (errors) must be random, normally distributed, with a mean of zero. It means that the difference between the model and the observed data should be close to zero. To verify it, two strategies were considered. (1) The Q-Q plot, or quantile-quantile plot, was created using qqnorm() and qqline functions from the ‘Stats’ package. No substantial deviations of the graph points from the diagonal indicate that there are no departures from normality. (2) We calculated the Shapiro–Wilk normality test using the shapiro.test() function from the ‘Stats’ package. The null hypothesis is that the errors are normally distributed. It is assumed if the *p*-value is greater than alpha, which was set in our study to 0.05.
  - The homoscedasticity assumption implies that the errors had a constant variance. That is, the errors are homogeneously distributed. This was verified via two procedures: (1) Graphically, representing the residuals as compared to the values adjusted by the model, and verifying that the former maintain a homogenous dispersion across the *x* axis. In other words, the residuals should roughly form a “horizontal band” around this. If there is any specific pattern, for example, cone-shaped or more dispersion at the ends, this means that there is no homoscedasticity. The ggplot() function in the ‘ggplot2’ package was applied. (2) Calculating the Breusch-Pagan test via the bptest() function, included in the ‘lmtest’ package. The null hypothesis assumes homogeneity of variance (homoscedasticity). This may be assumed if the *p*-value is higher than alpha (*p* > 0.05).
  - Independence of the errors’ assumption. This implies that the errors in the measurement of the independent variables do not reveal any first-order linear auto-correlation, i.e., they are independent from one another. It was verified through the Durbin-Watson test. Errors are considered independent if the Durbin-Watson statistic is found to be between 1.5 and 2.5. The dwt() function, found in the ‘car’ package, was applied
  - No collinearity. This assumption implies that the independent variables are not correlated to one another and therefore, there is no redundancy between them. On the other hand, when a strong correlation exists between the variables, there is multi-collinearity, and this is a problem. Two strategies were used to verify the lack of collinearity: (1) The correlation matrix between the independent variables: The corrplot() function in the ‘corrplot’ package was used. (2) The variance inflation factor (VIF): in general, it is assumed that values that are lower than 10 indicate no collinearity. However, other authors, such as Hair et al. [69], proposed more restrictive values (VIF < 4) to determine the lack of collinearity. The VIF() function, included in the ‘car’ package, was used.
  - Non-influential cases. To identify influential outliers in the set of independent variables, i.e., to detect points that negatively affect the regression models, Cook’s Distance was calculated [70]. The consensus is that a Cook’s Distance value of more than 1 indicates an influential value. The cooks.distance() function, included in the ‘base’ package, was used.

## 3. Results

The summary statistics of variables from each MLR model are presented in Table 1.

The MLR model testing Language AA was significant (F(3169) = 22.41, *p* ≤ 0.001). PA (*p* = 0.021) and gender, specifically, being female, (*p* ≤ 0.001), were positively associated with Language AA, whereas EF deficits (*p* ≤ 0.001) were negatively associated with it. These 3 variables accounted for 27–29% of variance in Language AA (R^2^ = 0.285, R^2^adj = 0.272). The magnitude of the β coefficients indicates that EF deficits (β = −0.461) had a greater weight in Language AA than PA (β = 0.160) and gender (β = 0.244). All of the assumptions of MLR (linearity, normality of distributed errors, homoscedasticity, independent errors, multicollinearity and no influential case) were met.

Mathematics AA was positively associated with PA (*p* = 0.097) and gender (*p* = 0.014) and negatively associated with EF deficits (*p* ≤ 0.001), explaining 19–21% of its variance (R^2^ = 0.214; R^2^adj = 0.195). For every additional point in PA (maintaining the other independent variables constant), Mathematics AA increased by 0.356 points. Being female also increased Mathematics AA (0.676 points). On the other hand, for each point in EF deficits, Mathematics AA decreased by 0.042 points. The magnitude of the β coefficients indicated that of the EF deficits (β = −0.435), the greatest weight was found for Mathematics AA and PA (β = 0.126) had the lowest weight. All of the assumptions of MLR were met.

In the case of Instrumental AA, the optimal MLR model included EF deficits, PA and gender (F(3169) = 20.73, *p* = 0.002). The association between EF deficits and Instrumental AA was negative (*p* ≤ 0.001). The association between PA and Instrumental AA was positive (*p* = 0.058). The association between gender and Instrumental AA (*p* = 0.002) was also positive. These 3 variables explained 26–27% of the variance in Instrumental AA (R^2^ = 0.269, R^2^adj = 0.256). All of the assumptions of MLR were met.

The MLR model testing English AA was significant (F(2170) = 20.23, *p* ≤ 0.001). EF deficits (*p* ≤ 0.001) were negatively associated with English AA, and gender (*p* = 0.021) was positively associated with it. Both variables accounted for 18–19% of the variance in English AA (R^2^ = 0.192, R^2^adj = 0.183). For every additional point in EF deficits (holding gender constant), English AA decreased by 0.039 points. For female participants (holding EF deficits constant), English AA increased by 0.636 points. According to β coefficients, EF deficits (β = −0.386) showed a greater weight in English AA than gender (β = 0.163). All of the assumptions of MLR were met.

In the case of Geography and History AA, EF deficits, PA and gender were included in the optimal model (F(3169) = 14.02, *p* ≤ 0.001), accounting for 19–20% of its variance (R^2^ = 0.199, R^2^adj = 0.185). The first variable (EF deficit) showed a negative association, and the others (PA and gender), positive. EF deficits (β = −0.353) had the greatest weight in Geography and History AA, followed by gender (β = 0.241). PA had a lower weight in Geography and History AA (β = 0.210). All of the assumptions of MLR were met.

The MLR model testing Physical Education AA was significant (F(2170) = 23.07, *p* ≤ 0.001). PA (*p* ≤ 0.001) was positively associated with Physical Education AA whereas EF deficits (*p* ≤ 0.001) was negatively associated. EF deficits and PA accounted for 20–21% of the variance in Physical Education AA. For every additional point in EF deficits (holding PA constant), Physical Education AA decreased by 0.020 points. For every additional point in PA (holding EF deficits constant), Physical Education AA increased by 0.423 points. The magnitude of the β coefficients indicated that EF deficits had a stronger weight in Physical Education AA than PA (β = −0.395 and β = 0.293, respectively). Linearity, independent errors, multicollinearity and non-influential case assumptions were met.

Overall AA was negative and significantly associated with EF deficits, and was positive and significantly associated with PA and gender (F(3169) = 19.65, *p* ≤ 0.001). For every additional point in EF deficits (holding the other independent variables constant), the Overall AA score decreased by 0.034 points. A one-unit increase in PA score (holding the other independent variables constant) was associated with an increase in the Overall AA score of 0.358 points. For female participants (holding the other independent variables constant), Overall score increased by 0.667 points. These 3 independent variables (EF deficits, PA and gender) explained 25–26% of the variation in Overall AA. EF deficits (β = −0.442) had the greatest weight in Overall AA, followed by gender (β = 0.226) and PA (β = 0.160). All of the assumptions of MLR were met.

In summary, all of the calculated MLR models were significant. In each of them (except the model testing English AA and Physical Education AA), EF deficits, PA and gender were included. Academic year was never included. In the model testing English AA, only EF deficits and gender were included. In the model testing Physical Education AA, only EF deficits and PA were included. As such, EF deficits were included in all of the models. This variable was always negatively associated with the AA. EF deficits always had the strongest weight in AA. The other variables included in the models, PA and gender (female), were always positively associated with AA.

## 4. Discussion

The goal of this study was to analyze if, in a sample of Spanish Compulsory Secondary Education students, the variables of EF, PA, gender and academic year contribute to the student’s AA (specifically, to their AA in each of the 5 common classes studied during all four of the years of Compulsory Secondary Education: Language, Mathematics, Geography and History, English and Physical Education, in addition to their Instrumental and Overall AA). The results obtained reveal that the level of the students’ EF and PA, in addition to their gender, partially determined their AA. However, this is not the case with academic year, which was not found to contribute to their AA.

The results obtained reveal the importance of EF in the student’s AA, regardless of whether this is considered for each of the analyzed classes, based on the mean of the instrumental classes, or is considered globally, in the five common classes of Compulsory Secondary Education. In all cases, the EF has been shown to be the variable that has the greatest influence on the student’s AA, so much so that difficulties in EF lead to a decrease in AA. The level of PA also influences the student’s AA (in this case, positively), except in the case of English AA, where it does not reveal any type of relationship. Therefore, engaging in PA leads to benefits in AA, except in the English class, where it does not affect it, either positively or negatively. However, in all of the tested models, PA contributed to AA to a lesser degree than EF. Being female is another variable that is associated positively with AA in all of the models, except in that of Physical Education AA. This suggests that female participants, maintaining the other variables as constant, obtained a higher AA than their male counterparts, for all of the classes, except for Physical Education (where gender did not appear to have an effect, given that there were no gender-based differences). The female participants also obtained higher mean grades than males in the instrumental classes (Instrumental AA) and in the mean of all classes (Overall AA). Being female, therefore, was found to be more important to AA than level of PA but is less relevant than EF. As previously mentioned, academic year was not found to have any type of significant association with any measurement of AA. Most of these results are in line with the current literature, although some differ from it, as detailed below.

The percentage of variability in AA that is explained by these 3 variables (EF, PA and gender) varied from R^2^adj = 0.183 to R^2^adj = 0.272, values found to be within an acceptable range in the social sciences, in general, and specifically, in the area of educational research [71,72,73]. AA is a multi-dimensional phenomenon and therefore it is impossible to consider all of the relevant and diverse variables participating. Therefore, explaining a large amount of its variation is a major challenge demanding considerable effort from a multidisciplinary research field [71]. In our study, we are aware of the variation of AA that remains unexplained in the models tested. Therefore, we believe that future studies should include new variables to further explain the student’s AA (either student variables or family and educational variables). The three variables that were included in the models here were found to be significantly correlated with AA. Therefore, major educational conclusions and implications may be derived from our results. These conclusions are presented below.

### 4.1. Contribution of EF to AA

The fact that EF is a predictor of AA has already been suggested in past literature. However, studies demonstrating the relationship between EF and AA have been mainly carried out using samples of children, with few studies considering this issue in adolescents [22,27,43]. Therefore, our work offers a major contribution in this sense. Our results indicate that deficits in EF (an aspect assessed using the BRIEF-2, in which high scores imply difficulties in the EF) are associated negatively with AA. As such, students with EF deficits obtained a lower AA than those students with a high level of EF, and this occurred in each of the individual classes, when considering the mean of the instrumental classes, or when considering the Overall AA. These results are in line with past empirical studies that show that the presence of EF deficits contributes to academic difficulties that may hinder AA success [74,75].

Given that EF includes the ability to direct one’s attention and behavior towards meeting a goal, these functions are necessary to carry out most academic tasks [76]. Therefore, regardless of the specific class that the student is in, academic work implies high demands for the student’s EF. It is necessary to plan study time, maintain one’s attention, inhibit distracting stimuli that are external to the task at hand, inhibit non-relevant information, select the most important information to recall and remember by using strategic revising techniques, relate information with other pieces of information, etc. Furthermore, especially in classes, it is necessary to carry out various tasks simultaneously: pay attention to and select the most relevant information that the teacher may be presenting via distinct means (visual and auditory), while at the same time, taking note of everything. All of these issues require the use of EF, and they are all necessary in the school context, regardless of the class and specific content being studied. Past research has indicated that students having good EF have better skills to benefit from the formal and informal learning opportunities that they are presented with, and therefore, obtain higher AA than students having poorer EF [77].

Thus, the results of our study (EF contributes to all AA measures) suggest an additional contribution to the limited information available on EF and AA in adolescents, pointing in the same direction as the findings for younger students. The results are in line with those of other authors, suggesting that EF is domain-general and required in various academic skills [22,27,43,74,76]. However, this does not allow us to reject the idea that EF is domain-specific, and that each EF component is related differentially to AA [78], given that, in the analysis performed, we did not consider the distinct components of EF separately. As we indicate below, this is one of the limitations of our study and is an issue that may be examined in future works. However, our work also offers advances given that it analyzed the relationship between FE and AA, beyond the fields of Language and Mathematics, including AA for other classes that had been yet to be analyzed.

Given that EF helps explain some of the variability of the adolescents’ AA, interventions that aim to improve EF should include improvements in AA. Therefore, increasing the students’ EF should be a goal throughout the educational context, even more so given that EF’s importance is not limited to its effect on AA but goes beyond this. A deficient level of EF during adolescence may lead to inadequate AA and to difficulties in facing and managing the physical, psychological and social changes occurring during this life stage [20,23,76]. Therefore, the importance of EF goes beyond the academic context to also affect the quality of life of the student and his/her ability to contribute to the progress of society. Since we know that during adolescence, EF are particularly sensitive to contextual influences and because interventions are more effective the earlier they are carried out, this life stage should be considered to ensure its improvement. Therefore, numerous activities and interventions are found to be effective in improving EF [79]. But there is still a lack of information as to which specific features of the training regimen have the greatest effects and on what type of participants, especially in the adolescent population, given that most of the studies in this area have focused on preschoolers. Therefore, we are unaware of the frequency, duration, intensity, etc., of the intervention in order to ensure the greatest effects, based on the specific characteristics of the individuals receiving the intervention: age, gender, motivation for the training tasks, initial FE level, etc. Additional fine-tuned analyses testing individual differences are necessary in order to determine for whom each training works.

### 4.2. Contribution of PA to AA

Many studies have corroborated our finding that PA is associated with AA. In all of the models tested in our study, except for the one referring to English AA, PA is positively associated with AA. The fact that no association was found to exist between PA and English AA suggests the need for additional research to explain this.

Most of the existing research analyzing the relationships between PA and AA do so on a global level, that is, without considering each of the academic domains. Few works have analyzed the specific associations existing between PA and AA in distinct academic domains, typically limited to language and, especially, to mathematics [29]. Furthermore, the results of these limited studies are quite disparate. Therefore, our results corroborate the findings of authors such as Katz et al. [41] and Álvarez-Bueno et al. [42] since associations were found between PA and AA in the language and mathematics fields, as well as for the composite AA grades. However, there are discrepancies with other works that only found associations between PA and some of the academic areas or, either language or math, but not both [35,39,80]. Other studies did not reveal any associations between these two areas [40,81]. Discrepancies between these studies may be due to distinct methodologies used (such as, for example, using academic notes or standardized tests to assess AA, as well as the assessment of distinct issues referring to PA: frequency, intensity, etc., with distinct measurement instruments), in addition to differences between the analyzed samples. Additional research is necessary to clarify this disparity of results.

We know of very few works that have analyzed the associations between PA and other academic areas, distinct from language and math. With regard to Physical Education AA, Poulain et al. [40] found positive associations between PA and AA in this class, with these results being in line with the findings from this study (although we have already mentioned that they did not find any associations between PA and Mathematics or between PA and Language). With regard to AA of a foreign language, such as English for our study sample, we are unaware of studies that have considered its association with PA, therefore we lack information to make comparisons and to explain the lack of a relationship found in our study. As we mentioned, additional research is necessary on this issue. However, we cannot confuse the lack of an association between PA and English AA with what may be a potentially inverse association. Therefore, it is important to note that engaging in PA, according to our results, is not associated with positive results in the academic area of English, nor is it associated with unfavorable results. As such, it is clearly necessary to carry out further research to explain this result.

The results of our study and past works should be taken into account by parents, professionals and policymakers who are responsible for the education and well-being of adolescents. These results suggest the importance of promoting PA amongst adolescents, given its benefits to AA. This is especially relevant if we consider that, despite the well-known benefits of PA, globally speaking, approximately 81% of all adolescents (between 11 and 17 years of age) do not reach the recommended levels: at least 60 min daily of moderate-to-vigorous intensity PA [28,82]. In Spain, the prevalence of adolescents that do not reach this recommendation is 76.6% [82]. Thus, less than one quarter of all Spanish adolescents comply with these recommendations. This situation is increasingly alarming given that the percentage of adolescents complying with the PA guidelines of the World Health Organization (WHO) is gradually decreasing, both internationally and nationally [82]. We are therefore far from reaching the target of a 15% relative reduction in insufficient physical activity among adolescents by 2030, established by the WHO member states [28]. Promoting and increasing PA in adolescents is urgent and necessary. It is known that during this life phase, the brain continues to be forming (as well as the adolescent) and is more sensitive to being influenced by the environment, therefore, increasing PA during adolescence would produce more benefits than in subsequent life stages [37]. In addition, adolescence is a key period of life in which individuals develop and consolidate habits that tend to persist over time. As such, if it is promoted during this phase, it is likely that the PA will be carried out throughout the individual’s life, with its inherent benefits [83].

Schools serve as excellent intervention settings to promote distinct daily PA actions in adolescents (and, therefore, an increase in their AA) given its educational nature and because this is where the adolescents spend the majority of their waking hours [83,84]. Of the PA carried out in the school context, Physical Education classes are the main component. In fact, these classes are often the only occasion for students to engage in PA [83]. It is necessary to put an end to the lower status given to Physical Education, as compared to other academic subjects, and to value its important role in improving not only the students’ health but also their AA. Physical Education classes should be highlighted as an important means of fostering PA habits in young people and increasing their AA. Thus, educational policies which, at specific times, have attempted to impose and decrease the number of hours dedicated to Physical Education, in favor of more hours devoted to instrumental classes, were not supported by scientific evidence. Many studies have indicated that spending more time in Physical Education classes, even when this means spending relatively less time on other school subjects, does not jeopardize AA, but possibly even improves it [35]. Spain is one of the countries in which fewer hours of the school curriculum are devoted to Physical Education. In 2013, the Eurydice [85] study warned that in Spain, the Physical Education class made up only 3% or 4% of the Compulsory Secondary Education curriculum, far from the 8% that is recommended by the American Heart Association and even farther from the almost 20% of Finland (a country having top results in the PISA). This may contribute to understanding the poorer performance of Spanish students in international assessments such as the PISA.

Other compatible measures to increase PA within the school context and therefore, to improve the AA of students, is to increase PA in other school areas and times, distinct from the Physical Education class. This may be seen by introducing active games and providing sports equipment during recess, including short bursts or bouts of physical activity between lessons, either with curriculum content (curriculum-focused active breaks) or without (active or dynamic breaks), or creating physically active academic lessons (to incorporate PA into the teaching of academic lesson content) [34,86]. At times, however, these practices (especially physically active academic lessons) may face certain barriers, such as overly traditional educational policies or a lack of teacher training to carry them out, especially in the Secondary Education level [86]. Work should be carried out in this sense.

It is also important to promote the practice of PA in associations, clubs and entities existing outside of the school, since this will contribute to the adolescents’ continued practice of PA, even outside of the school setting. This will help ensure that the multiple benefits of PA extend beyond this life phase.

### 4.3. Contribution of Gender to AA

Another variable that has been found to be significant in all of the models tested in our study, except in that of Physical Education AA, is gender. Being female was positively associated with AA in all of the classes except for Physical Education, as well as in the Instrumental and Overall AA. In Physical Education AA, gender was not found to be a significant variable. These results are somewhat coherent with the existing literature and with the data resulting from national and international assessments. However, this is not in all aspects. Our results are in the same direction as those of the national and international literature suggesting a higher Overall AA in female students, as well as in Language and other classes in which verbal skills are especially involved, such as English as a foreign language [11]. However, our results are not coherent with these in that we did not find that the female gender was also positively associated with Mathematics AA. Although in most of the past research, and in the international assessments, boys are found to have higher Math AA than girls, there are other studies that have distinct results in which girls obtain higher Math AA [87] or that do not reveal significant gender-based differences [49].

Very few studies have analyzed the effect of gender on classes other than Language and Math, with a gap existing on this area which our study attempts to fill. Thus, our work finds that in the lesser studied classes, such as History and Geography, being a woman contributes positively to one’s AA, and in Physical Education, it does not have either a positive or negative effect. In our results, gender does not appear to contribute to Physical Education AA. This aspect differs from the findings from other studies, where quite discrepant results have been found. As such, for example, González Hernández and Portolés Ariño [50] found that males had better AA in Physical Education, while in the study by Pellicer-Chenoll et al. [88], girls were found to have better Physical Education AA. Diverse aspects, such as the content of Physical Education assessed in each study, may cause these disparate results.

According to the literature, biological and socio-cultural factors should be considered in order to understand the mechanisms by which gender may affect the student’s AA. Both types of factors, biological and socio-cultural, act on the interaction and cannot be separated [40]. Positive association between being female and AA, as found in our study, may not be explained by the different levels of testosterone between genders (since, according to many studies [46,47], the higher level of testosterone in males favors their visual-spatial abilities, leading to an expected association between being male and Mathematics AA, a result that was not found). However, other studies indicate other effects of testosterone that may explain the results of this study. Higher testosterone levels are associated with decreased capacity for learning [89]. This finding may contribute to explaining our results in which males have a lower AA in all of the classes except for Physical Education, in which gender had no effect on AA. In this same direction, other studies have indicated that females (having lower testosterone levels) have a higher self-regulation level, helping them to adapt more to the learning tasks and school demands. These studies have also found increased ability to concentrate, more hours dedicated to studying, increased compliance with rules, etc. Ultimately, females have more adaptive behavior facilitating learning and fewer disruptive behaviors, clearly helping to give them a higher AA. It appears, therefore, that certain neuroendocrine differences between the genders may lead to distinct behavioral patterns at school, which may be the basis of certain theories that have suggested the faster and easier ability of females to align with expectations and norms of the society, in general and especially in school [90]. Thus, there are links between the endocrine system, the brain and behavior that are relevant to understanding adolescent AA. However, these links are not completely understood [90], given that these links may also be affected by socio-cultural factors. Along these lines, the literature highlights the importance of socialization processes and, especially, the possible gender stereotypes existing in the context in which the adolescents develop, when explaining gender differences in AA. So, we consider that in order to explain our results, especially the positive association found between being female and Mathematics AA (a result that differs from many findings of past studies and international educational assessments), it is important to consider the educational policies implemented in Spain that go against these traditional gender stereotypes.

For several years, Spain has been working on social and educational policies regarding equality, as well as female empowerment policies in an attempt to deconstruct the traditional gender stereotypes and to increase the interest of girls in STEM studies. These policies are proving successful, as shown by this study, with the positive association found between being female and Mathematics AA. Some national data points to the results of the PISA, in which the gender gap found in Mathematics continues to gradually decrease [45]. Other studies have highlighted the effects of these actions: to decrease traditional gender-based stereotypes (i.e., boys are better than girls at math) and to increase the interest and performance of females in these STEM areas [91].

### 4.4. Contribution of Academic Year to AA

Our results referring to the lack of association between academic year and AA do not agree with data from other national and international assessments [7,45], where AA decreases as the lower secondary education year increases. The results are not coherent with certain studies that suggest that the youngest high school students obtain the highest mean scores on AA, while the older students had the lowest mean [52]. The differences between our results and those from this series of works may be caused by the orientation and mentoring actions carried out in the participants’ school. It was indicated that the school-based factors (such as implementation of orientation and mentoring programs) make up another series of variables (not considered in this study) which contribute to the students’ AA. The mission of the school itself focuses on providing students with an enriched and supportive academic environment. Thus, the school attended by the participants notified us that since the year prior to this study, it has been participating in an ambitious guidance and mentoring program that involves not only students but also their families and the entire school teaching staff. It is possible, therefore, that the AA of these students would be different if the students had not received this program. It may be necessary to determine what happened with other students coming from other schools in which said program was not carried out, or not carried out as intensely as in this case.

### 4.5. Limitations

The results from this study offer an improved understanding of the impact of these different factors (EF, PA and gender) on the students’ AA. Despite the implications for the educational field, the results should be considered with caution, given that the study has certain limitations that are described below. These limitations should be taken into consideration in order to overcome them in the future.

First, the size and type of sample studied: the sample was relatively small and was not obtained probabilistically. The inclusion of participants from only one school makes it difficult to generalize the results. It would be interesting to expand the study to a larger sample size coming from distinct schools that are randomly selected. For example, to include more schools with different characteristics, i.e., private, concerted and public schools, and rural, urban and suburban would be interesting, and in addition, it would allow to know if there are differences between these groups in AA.

Second, due to time constraints, the study design was cross-sectional; therefore, no causal inferences can be made. Future studies should use a longitudinal design to examine the causal relationships.

Third, data were collected using self-reporting methods or via information from third party informants. Although they are well-proven assessment methods in different domains of social, emotional and behavioral functioning [92,93], some limitations are found. To overcome the limitations of this type of measures, it would be interesting to complement the study using distinct data collection methods. This would determine additional information for each assessed variable. In this study, the School version of the BRIEF-2 was used to assess the participants’ EF. It is an informant reported questionnaire designed to index children’s everyday EF skills. Its good–excellent psychometric characteristics have been reported by many studies [52,92], turning into the most used informant reported questionnaire to evaluate executive functions in adolescents [92,94]. However, it should not be forgotten that this is a rating scale, and although many defend the use of this type of instruments, many others warn of their limitations (social desirability and the assessment of behaviors that are not the subject of the study but the informant’s perception of the same) [95]. For this reason, it may be interesting to complement their use with other measures that are traditionally considered to be more objective, such as performance-based tests. These measures also have their limitations, however, such as a limited functional and ecological validity [96], since performance-based assessment of EF often occurs in a one-on-one setting with fewer distractions than the typical environment. In addition, existing evidence on psychometric robustness of such performance-based measures remains limited. For this reason, we consider that an excellent alternative would be the observations of every-day, real-world, executive functioning behaviors. The use of observational coding systems does not require reporting from important adults in the students’ life. Although this implies certain costs, especially time-related ones, the quality and depth of the information collected as well as its functional and ecological validity is undeniable [97]. On the other hand, to assess the level of PA, a self-reporting method was used. Although the instrument used (PAQ-A) has adequate psychometric properties and its use has been contrasted with other instruments such as accelerometers, the inherent limitations of self-reporting are well-known, raising the question of social desirability bias as well as the difficulty of some participants in informing about their own behaviors, cognitions and feelings [95]. Therefore, we should not discard the presence of these biases in the participants’ responses, especially the social desirability bias, since it is possible that our participants exaggerated their level of PA. In the future, to contrast this, it may be interesting to complement this information collection measure with other measures, such as accelerometry. But these instruments have their own limitations, such as the impossibility of their use in aquatic activities and their limited effectiveness in assessing static physical activity, and we cannot forget that when using accelerometers, participants are aware that they are being assessed and measured and therefore may alter their normal level of PA [98]. Of course, we should consider the economic costs of the same (even higher when taking into account that the goal is to assess the PA of the entire sample during the same week to ensure that PA patterns are not affected by other factors, such as different weather conditions).

As previously mentioned, although the most common method, the use of academic grades as an indicator of AA has its limitations (the possibility of being affected by subjective aspects of the teachers, as well as level of demand, years of experience, etc.). However, despite these limitations, many authors have highlighted the utility of this method [3]. In Spain, school grades that are issued by teachers are very important in daily educational practice. Decisions regarding the student’s school path tend to be based on these grades. Therefore, the use of assessment measures that are commonly used in school practice may maximize the applicability of the study findings [99]. It may be interesting, however, to replicate the results using other AA indicators such as standardized test scores. We should note that this may require considerable technical, human and financial resources, which are not always available.

### 4.6. Strengths

As previously mentioned, some of the highlights of this study are: (1) it jointly includes the variables EF, PA, gender and academic year and their relationship to AA in a single study. These variables, as far as we know, had not been previously studied together, especially in adolescents. (2) It considers an extensive perspective of adolescent EF that includes its hot and cool components (and not only the latter, as typical of most studies), and (3) it considers an extensive perspective of AA, by specifically considering it in all of the subjects that are common to the 4 school years of Compulsory Secondary Education, in addition to examining AA based on the instrumental classes and overall AA. This required studying more than just Language and Mathematics, which have been the focus of most past research. In future studies, it would be interesting to delve further into the specific relationships between all of these variables. For example, to analyze the contribution of the distinct subcomponents of the cool and hot EF in each of the classes. Although it appears that not all subcomponents of EF are of equal importance in learning and AA in the distinct academic areas, researchers have yet to offer conclusive results, at least for adolescent samples [43]. More studies are necessary on this aspect. The same could be analyzed in terms of PA. In this work, we have considered the level of PA carried out by students, but it would be interesting to examine whether or not different aspects related to PA (force—resistance and explosive, agility, speed, aerobic endurance, flexibility, etc.) contribute differently to AA in each class and the skills required by the same. Past research appears to indicate this, but further studies are necessary [43].

We believe that the objectives of future works should include not only the analysis of specific multiple relationships but should also consider the questions of how and why. This requires extensive and complex multidisciplinary research that requires support of governments and their policies. It should be understood that all investments in improving student AA will ultimately have positive effects on society.

## 5. Conclusions

The results of this study shed light on the differential contributions of EF, AP and gender on the AA of Spanish Compulsory Secondary Education students, considering it both specifically for the five distinct subjects that are common to this educational period (Language, Mathematics, Geography and History, English and Physical Education), as well as the mean of the instrumental or general classes. The results also revealed that academic year does not affect the student’s AA (regardless of how this is considered). These results offer information that is relevant for the design of interventions that would improve student AA, and this should be a priority for all governments, since the academic level of their citizens is associated with the progress of the nations.

## Figures and Tables

**Table 1:** Summary statistics of variables from each calculated MLR model.

Dependent Variables	Independent Variables	*B*	*SE B*	β	95% CI		*t*	*p*	*Multiple* *R^2^*	*Adj.* *R^2^*	*F*
Language AA											
	Intercept	7.442	0.823		5.817	9.068	9.040	<0.001 ***	0.285	0.272	220.41
	EF	−0.040	0.006	−0.461	−0.051	−0.028	−6.986	<0.001 ***			
	PA	0.404	0.173	0.160	0.062	0.745	2.334	0.021 *			
	Gender ^a^	0.810	0.229	0.244	0.358	1.262	3.541	<0.001 ***			
Mathematics AA											
	Intercept	7.564	1.024		6.117	9.991	7.385	<0.001 ***	0.214	0.195	11.41
	EF	−0.042	0.007	−0.435	−0.052	−0.026	−6.039	<0.001 ***			
	PA	0.356	0.213	0.126	−0.142	0.671	1.671	0.097			
	Gender ^a^	0.676	0.272	0.181	0.116	1.193	2.486	0.014 *			
English AA											
	Intercept	8.642	0.764		7.133	10.153	11.299	<0.001 ***	0.192	0.183	20.23
	EF	−0.039	0.007	−0.386	−0.053	−0.025	−5.542	<0.001 ***			
	Gender ^a^	0.636	0.272	0.163	0.099	1.173	2.340	0.021 *			
Geography & History AA											
	Intercept	6.032	1.047		3.965	8.098	5.761	<0.001 ***	0.199	0.185	14.02
	EF	−0.037	0.007	−0.353	−0.051	−0.022	−5.062	<0.001 ***			
	PA	0.634	0.220	0.210	0.200	1.069	2.884	0.004 **			
	Gender ^a^	0.962	0.291	0.241	0.388	1.536	3.306	0.001 **			
Physical Education AA											
	Intercept	8.409	0.379		7.662	9.156	22.215	<0.001 ***	0.213	0.204	23.07
	EF	−0.020	0.003	−0.395	−0.026	−0.013	−5.775	<0.001 ***			
	PA	0.423	0.099	0.293	0.227	0.618	4.270	<0.001 ***			
Instrumental AA											
	Intercept	7.748	0.834		6.102	9.394	9.295	<0.001 ***	0.269	0.256	20.73
	EF	−0.040	0.006	−0.457	−0.051	−0.028	−6.863	<0.001 ***			
	PA	0.334	0.175	0.132	−0.011	0.680	1.909	0.058			
	Gender ^a^	0.732	0.232	0.221	0.275	0.680	3.161	0.002 **			
Overall AA											
	Intercept	7.520	0.745		6.049	8.991	10.090	<0.001 ***	0.259	0.245	19.65
	EF	−0.034	0.005	−0.442	−0.044	−0.024	−6.587	<0.001 ***			
	PA	0.358	0.157	0.160	0.048	0.667	2.284	0.024 *			
	Gender ^a^	0.667	0.207	0.226	0.258	1.076	3.222	0.002 **			

AA = Academic Achievement, EF = Executive functions; PA = Physical Activity; *B* = Unstandardized beta; *SE* = Standard error for the unstandardized beta; β = Standardized beta or standardized regression coefficient; CI = Confidence interval; *t* = t-statistic value; *p* = p-value of t-statistic; *Multiple R^2^* = Multiple R-squared; *Adj. R^2^* = Adjusted R-squared; *F* = F-statistic value; ^a^ Male = 1, female = 2; *p* < 0.1, * *p* < 0.05*,* ** *p* < 0.01, *** p < 0.001. MLR: Multiple Linear Regression.
